# Annual Transmittance Behavior of Light-Transmitting Concrete with Optical Fiber Bundles

**DOI:** 10.3390/ma16217037

**Published:** 2023-11-04

**Authors:** Adithya Shenoy, Gopinatha Nayak, Adithya Tantri, Kiran Kumar Shetty, Mangeshkumar R. Shendkar

**Affiliations:** 1 Department of Civil Engineering, Manipal Institute of Technology, Manipal Academy of Higher Education, Manipal 576104, Karnataka, India; adithya.shenoy@learner.manipal.edu (A.S.); kiran.shetty@manipal.edu (K.K.S.); 2 Department of Civil Engineering, Manipal Institute of Technology Bengaluru, Manipal Academy of Higher Education, Manipal 576104, Karnataka, India; shendkar.mk@manipal.edu

**Keywords:** light-transmitting concrete, daylighting, illumination, optical fibers, transmittance, ANOVA

## Abstract

This study characterizes the transmittance behavior of structural light-transmitting concrete under natural sunlight. The experimentation involves the use of a novel test setup and a detailed analysis considering the variation and dependence on time of day, month of the year and seasonal variations. The test set consisted of 28 variations of fiber configurations, with two different diameters, spacing and bundling techniques used to increase the area of fibers while maintaining spacing to aid the placing of concrete without compromising on transmittance. The study provides a real-time observational understanding of the behavior of light-transmitting concrete, a result usually obtained by modelling and simulation. The statistical analysis helps in understanding the impact of various variables as well as their interrelationships, which can help in design optimization. Based on the behavior as well as the stipulations of standards, the applicability of the material to various structural applications has been identified.

## 1. Introduction

The evolution of concrete has given rise to innovative designs that transcend mere aesthetics and serve both functional and ecological purposes. Once confined to its role as a load-bearing material, concrete has transformed into a specialized substance with distinct properties, encompassing setting, hardening and durability. This adaptability, attained through meticulous molecular-level modifications, has elevated concrete to one of the most prevalent and reliable construction materials globally. As the world increasingly embraces environmental consciousness and sustainability, the imperative to develop environmentally friendly construction materials and processes that safeguard energy and the ecosystem has grown exponentially.

A noteworthy endeavor in this direction is the advent of light-transmitting concrete, ingeniously harnessing sunlight to curtail energy consumption for illumination while elevating the visual allure of structures. This groundbreaking concept, popularly known as LiTraCon, originated in the early 2000s through the visionary craftsmanship of Hungarian architect Aron Losonczi. Pioneering researchers have tirelessly explored an array of materials and chemicals to achieve translucency in concrete panels, using polymeric and siliceous optic fibers as key components and inventing construction materials that are both energy efficient and aesthetically captivating. Optic fibers have been embedded in concrete, in combination with other fibers such as glass, in order to enhance the flexural properties of concrete. The use of glass fibers enhances the split tensile and flexural strength of concrete with unidirectional optic fibers [1]. The inclusion of these fibers comes with three major parameters, namely spacing, diameter and number of fibers. Fiber spacing has been found to have a detrimental impact on transmittance mostly as increased spacing in turn indicates reduced area of transmittance per total area of concrete [2,3].

Amidst the backdrop of a global energy crisis, scholars have fervently pursued renewable energy alternatives to supplant finite resources. For instance, the surge in total energy consumption has been considerable in India, with a substantial share devoted to illuminating residential, commercial and industrial sectors [4]. Light-transmitting concrete presents a solution to mitigate this energy burden. Moreover, human well-being stands to gain significantly from exposure to natural light as research unequivocally demonstrates that daylighting enhances productivity and alleviates the perceived challenges of work environments while having both physiological and psychological impacts when juxtaposed with artificial lighting. Studies by pathologists and biologists have proven that sunlight strongly boosts the immune system against influenza, especially the H1N1 epidemic [5]. Sunlight also triggers natural processes and seasonal changes while modulating mood, stress and education in humans [6]. Psychological disorders such as bipolar disorder have also been proven to have later onset when there is exposure to sunlight, and mood related disorders have been found to reduce both in the intensity of their affects as well as the frequency of occurrence in patients from countries and regions with better sunlight exposure [7]. Studies have also shown that pre-natal sunlight is one of the most significant determinants on the size and health of infants and this pre-natal sunlight has an impact on the height of humans for life, measured until the age of 26 [8]. Apart from these physical and psychological impacts, even our perceived happiness and sadness is dependent on natural light and daylighting [9].

Numerous research endeavors have been dedicated to developing cementitious materials that facilitate light transmission. Researchers have established correlations between the area of fibers and light transmittance, encompassing various fiber volume fractions and diameters. Materials like waste glass, resin, optical fibers, translucent powders and other materials have been used as transmitting members in light-transmitting concrete with their own advantages and limitations [10]. The influence of the angle of incidence on transmittance of light transmitting concrete has been studied and the direct incidence of light is found to have the best transmittance. Artificial luminaries at angles of 30° or higher seem to have low transmittance characteristics and the other factors, such as the number of fibers or their diameter, seem to be of low significance especially at higher incidence angles [11]. Translucent concrete walls exhibit a climate-responsive transmittance characteristic, effectively optimizing solar heat gain during winter through increased transmittance while minimizing heat gain during summer by reducing transmittance. This dynamic performance is particularly advantageous in regions with middle to high latitudes, offering a superior ability to cater to varying seasonal solar heat gain requirements when compared to conventional building envelope systems [12]. The use of phase change materials as a thermal insulating and translucent sandwich material as a building façade has shown how translucence can be a property that can influence energy characteristics both for insulation and transmittance, especially in high altitude regions [13].

Simulation studies have been diligently executed to appraise the performance of light-transmitting concrete when subjected to natural light conditions. A simulation of the daylighting performance of translucent concrete building envelopes has elucidated how the fibers with larger numerical apertures, like 0.7, compared to smaller ones, like 0.5, can enhance average luminance flux up to 40.62% [14]. A numerical analysis of translucent photoluminescent building envelopes has substantiated the benefits of the use of translucent materials as an effective means to harness natural energy and reduce the energy demand of a building for lighting [15,16]. A study on the optimization of fiber dosage for translucent concrete building envelopes, focusing on the thermal and lighting energy savings, have indicated that the use of 5.6% fiber volumetric ratios can reduce energy costs by 18% [17]. The impact of the use of different transmitting media, various mineral and chemical additives, advanced casting and construction practices and analytical tools have been employed to estimate the behavior of this material. Waste glass has been used as an inclusion in manufacturing thin, translucent and photocatalytic concrete wherein limitations were observed in translucence based on the shape and size of the included glass [18]. Such concrete has also been studied from the perspective of the material being a prospective solution for photovoltaic roads [19]. Acrylic sheets in combination with glass have also been used in the manufacturing of ecological light-transmitting concrete, wherein a MATLAB analysis indicated how the materials when constructed as blocks could achieve high transmittance of up to 16% and be classified as a green building material [20]. Resin translucent cement mortar has also been studied as a means to replace bricks. The epoxy resins enhance compressive strength in small doses while increasing translucence and rationalizing energy expenditure [21]. A detailed analysis of the thermal behavior of light-transmitting concrete indicates how thermal transmittance has an insignificant impact on the air-conditioning costs and proving the efficiency of the material as an insulator [22].

While having a database on these characteristics, the present research scenario lacks information regarding real-time data on the material exposed to natural light. The volatile nature of the atmosphere and weather means modelling and simulation produce similar results but ignore outliers that might not necessarily conform to modelling conditions.

This comprehensive study aspires to elucidate the behavior of light-transmitting concrete under natural light conditions, encompassing the intricate interplay of environmental factors on light transmittance. The research delves into the influence of diurnal variations, seasonal dynamics, monthly fluctuations and annual trends on transmittance characteristics. In addition, the study endeavors to identify the most appropriate applications of diverse variants per the stipulated guidelines of Indian Standards.

One of the scientific terms used in the manuscript are transmittance, which is the percentage ratio of transmitted light to incident light. The loss in transmittance is the difference between the amount of light entering and exiting the transmitting media, mainly caused due to absorption and scattering or reflection at the surface of the fiber. Illumination, or illuminance, is the amount of luminous flux per area at any point on a surface.

### Research Significance

This research focuses on exploring the possibility of using light-transmitting concrete as a structural concrete, which would be a material that would add to the list of green energy materials that are the need of the hour. The work is a detailed experimental investigation into transmittance behavior and presents the results of 1 year of data collection and subsequent analysis in order to understand the effects of factors such as time of the day and weather conditions. The data reflect how there is a variation in behavior based on weather and a detailed statistical investigation using multivariate analyses of variances sheds light on what specific factors impact certain behaviors in the concrete. Additionally, the manuscript also highlights an experimental methodology using specifically designed equipment. The interdependence of various factors on transmittance, the influence of these factors and a possible method to optimize the inclusion of fibers based on diameter, spacing and the total number of fibers used per bundle is possible and can form the base for further investigation into prototypes or durability characteristics, either of which can help in realizing the material and accelerate its integration into the infrastructure and construction industry.

## 2. Experimental Methodology

### 2.1. Materials and Properties

The concrete was produced using OPC 43 grade, well-graded river sand, coarse aggregates with a maximum grain size of 10 mm, fly ash, potable water and a superplasticizing agent. The materials were tested for quality based on the stipulation of Indian Standards. Cement conformed to IS 8112:2013 [23], aggregates to IS 383:2016 [24] and fly ash to IS 3812 (Part 1): 2013 [25].

### 2.2. Optical Fibers

Optical fibers of large numerical aperture and acceptance cones were selected for the study. Polymeric bare fibers with an acceptance angle 61° and a numerical aperture of 0.51 and of two diameters, namely 0.5 mm and 1 mm, were procured from M/s Edmund Optics, India. The fibers had polymeric cores made of PolyMethyleneMethAcrylate (PMMA), colloquially known as acrylic fibers. The fibers were chosen to have a large acceptance angle since the source of light was sunlight and hence highly scattered. The optic fiber was examined under an SEM to inspect and understand the nature of the longitudinal and cut surfaces. The specific gravity, tensile strength and modulus of elasticity of these fibers were 1.18, 38.36 MPa and 95.60 MPa, respectively.

#### SEM Result Analysis

Scanning Electron Microscopy is an essential tool to analyze the surface morphology and size distribution of particles in concrete. The surface properties affect how the particles interact in the matrix and can give indications of the behavior of the constituents in the matrix.

**Figure 1:**
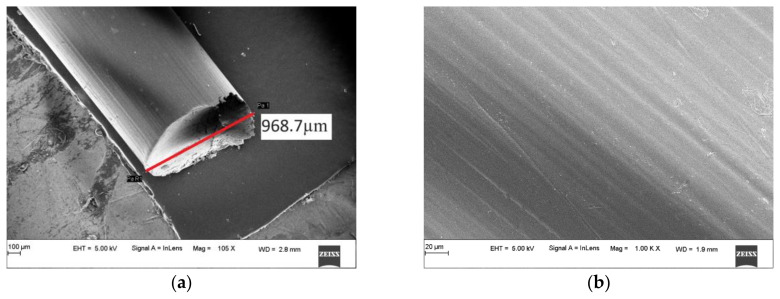
SEM results of optical fibers: (**a**) cut surface, (**b**) longitudinal surface.

The SEM analysis of optical fibers was carried out to determine the cut surface properties and the surface properties. The surface impacts the bonding efficiency, with rough surfaces conducive to better bonds, whereas smoother surfaces reduce the bond strength. More irregular surfaces impart better friction and pull-out resistance as they increase the surface area available for bonding and help with interlocking. The cut surface properties are essential to the study as they can affect the transmittance properties, and the technique used to cut fibers can result in high losses in transmittance if the surface is uneven. There can also be reflective and refractive losses due to rougher surfaces. Figure 1a is a typical cut surface, and Figure 1b is the typical longitudinal surface of a fiber. The cut surface indicates that the fiber has no crushing, with a diameter of 0.982 mm for the 1 mm of fiber. Other such samples also had variations between 0.978 mm and 0.994 mm, within acceptable manufacturing limits of error. The visual inspection of the surface also shows a mostly flat and clean cut, with pinching at one edge. This indicates a low loss in transmittance, as evident from the results of transmittance tests. The longitudinal surface in Figure 1b shows that the fibers have a relatively smooth surface, which could lead to pulling out and losses in strength. Still, the loss is very low in most cases due to the relatively small diameter of the fibers compared to the total area of concrete in which it is embedded.

### 2.3. Mix Design and Designation

**Figure 2:**
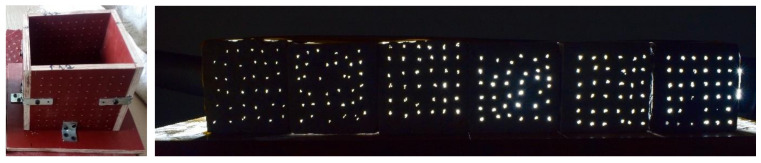
Special molds and light-transmitting concrete with different fiber bundle sizes.

The chosen type of concrete that was suitable for the closely spaced fibers in the mold was Self-Compacting Concrete (SCC). SCC is known for its high workability and passing ability, and the closely spaced fibers behave as a highly reinforced section, thus it is the best-suited type of concrete. The design of concrete such as SCC provides flexibility and the required workability while opening options to improve fresh properties through gradation techniques while maintaining strength [26,27]. The concrete mix design was created per IS:10262 and EFNARC 2005 [28,29]. The concrete was poured into pre-woven molds that contained the fibers preplaced in them, as discussed in the forthcoming section and indicated in Figure 2. These were then allowed to set and harden before the edges were trimmed. Twenty-eight variants based on the number of fibers per bundle, fiber diameter and spacing were cast and tested. The maximum number of fibers per bundle was optimized based on the strength requirements of concrete. The sample designations and their indicative symbols are given in Table 1, and the concrete mix proportions are in Table 2.

### 2.4. Mold Preparation, Casting and Specimen Preparation

The fabrication of light-transmitting concrete requires specialized molds capable of integrating fibers during the casting process. These molds play a crucial role in securing the fibers in place during casting, ensuring that their ends remain exposed to light for effective light transmittance, as illustrated in Figure 2.

Conventional industrial casting techniques involve layering thin alternating layers of mortar and fibers using mechanical means. However, when applied to concrete, this method proves inadequate due to coarse aggregates, causing the fiber ends to become embedded within the concrete and resulting in significant light transmission loss. Researchers have adopted innovative approaches to address this limitation, such as utilizing unique molds and weaving techniques to incorporate fibers within the concrete [30,31,32].

In this particular study, the researchers employed perforated molds measuring 100 × 100 mm. The spacing of the perforations adhered to the guidelines specified in IS 456:2000 [33], which dictate that the minimum spacing between reinforcements should be two-thirds the maximum size of the coarse aggregates or 15 mm, whichever is greater. Since the maximum aggregate size, in this case, is 10 mm, the minimum spacing chosen was 15 mm. Additionally, given the flexibility of the fibers, the researchers experimented with two different spacing variations in the concrete mix: 15 mm and 12 mm.

The casting process involved two main steps: weaving the fibers and pouring the concrete. The fibers were individually woven into the molds based on predetermined bundling sizes. After completing the fiber weaving, SCC was mixed and poured into the molds. Since SCC does not require compaction, the samples were lightly compacted using a table vibrator due to the close spacing of the fibers. The samples were then left to set and harden. Once sufficiently hardened, the ends of the fibers were trimmed and the samples were de-molded before ponding for curing. After reaching desirable strength and setting, the fiber ends were cut precisely and a hand grinder was used to achieve a smooth and uniform surface suitable for testing purposes.

### 2.5. Optical Properties under Natural Light

**Figure 3:**
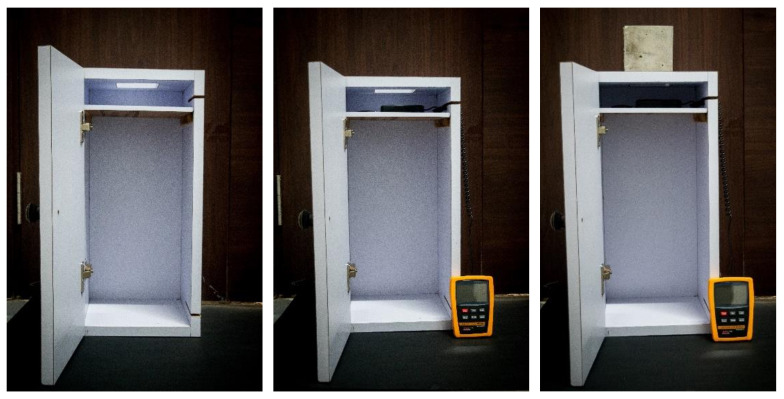
Vertical lightbox to measure the transmittance of natural light.

Optical properties were measured with specially designed equipmentThe equipment consisted of a black box with an aperture at the top to rest the cube and a mechanism to rest the receiver of the lux meter within the black box, as shown in Figure 3. The lux meter used was an LX-101A manufactured by M/s Hazari Tech Connect, Mumbai, India. The box was then placed in direct sunlight, and the intensity of light being allowed to transmit was measured in lux. Readings were taken at 0.05 m, 0.5 m and 1 m distance from the face before being averaged. The main focus was providing light during the working hours of most offices and educational institutions, which is 09.00–17.00 h, which is why luminance was captured at 1 h intervals between 09.30 and 16.30 h. Such readings were taken for the duration of 1 year at 5-day intervals to determine the effects of time of day, monthly variations, seasonal variations and an annual average of transmittance [14,17,34,35,36,37]. The experiments were carried out in Manipal Institute of Technology Campus in Karnataka, India (13.3525° N, 74.7928° E).

### 2.6. Statistical Analysis

The transmittance results depend on multiple factors like the incorporation of fibers, the number of fibers, the area of fibers, spacing, time of the day, month, season, etc. Analysis of variance (ANOVA) was carried out to determine and validate the results obtained. ANOVA is a powerful statistical tool that helps determine the effects of independent variables on the dependent variables. It also gives a clear picture of the influence of a combination of these independent variables on the test parameters. Understanding the magnitude of the effect these parameters have and the significance of these variables on our test parameters is essential in understanding and customizing the material based on the purpose of use. ANOVA is a hypothesis testing tool where the testing is conducted to determine the effect of one parameter on another. The *p*-value of ANOVA results indicate the level of significance based on which the null hypothesis is rejected or accepted. The null hypothesis of ANOVA is usually that the parameters have no significant impact on each other. The testing was performed based on a 95% confidence interval, based on which the *p*-values have been accepted or rejected, and the significance of an independent variable on a dependent variable has been determined [38]. Since the study involves over three independent and dependent variables, a Multivariate Analysis of Variance (MANOVA) approach was adopted. MANOVA is used in specific cases where more than one independent variable can impact the dependent variable simultaneously, and there is an expected interplay of these independent variables on the dependent variable.

## 3. Results and Discussions

### 3.1. Depending on the Time of Day

A plot of the annual illumination average vs. time of day illustrates the dependence of the time of the day on luminance. As the angle of incidence of sunlight reduces, there is an increase in the transmitted intensity of luminance. All mixes show maximum luminance at 12.30 p.m., and the midday durations exhibit higher luminance than the ends of the day.

**Figure 4:**
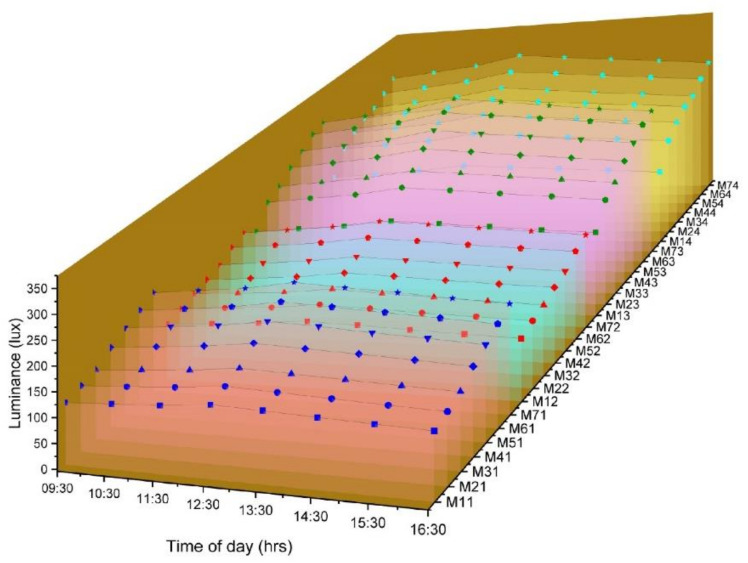
Variation of luminance based on the time of the day, for different samples from M11 to M74.

In the current study, the variable chosen for analysis is the average annual illuminance, illustrated in Figure 4. The independent factors influencing these were the time of the day, the diameter of fibers, the number of fibers and fiber spacing. Research has shown that transmittance is affected by the number of fibers more than their diameter. These indicate an interaction between these parameters, which has been investigated employing MANOVA analysis.

The illumination values that were obtained over the year were compiled and averaged relative to the time of measurement, which was then considered as the average annual illumination. The impact of various factors on the average annual illumination is represented in Table 3. The MANOVA was conducted with a 99.5% level of significance. The statistical results indicate a direct effect of the time of testing, diameter of fibers, fiber spacing and number of fibers on illumination. The simple main effect of each variable on lighting is significant as the *p*-values are below 0.005.

The interaction effects of Time and Diameter, Time and Spacing, Time and No. of Fibers, Diameter and Spacing, Diameter and No. of Fibers, Spacing and No. of Fibers as well as the effect of the combination of Diameter, Spacing and No. of Fibers on the illumination were also studied. The statistical analysis clearly shows that each parameter has an individual and combined influence on illumination.

**Figure 5:**
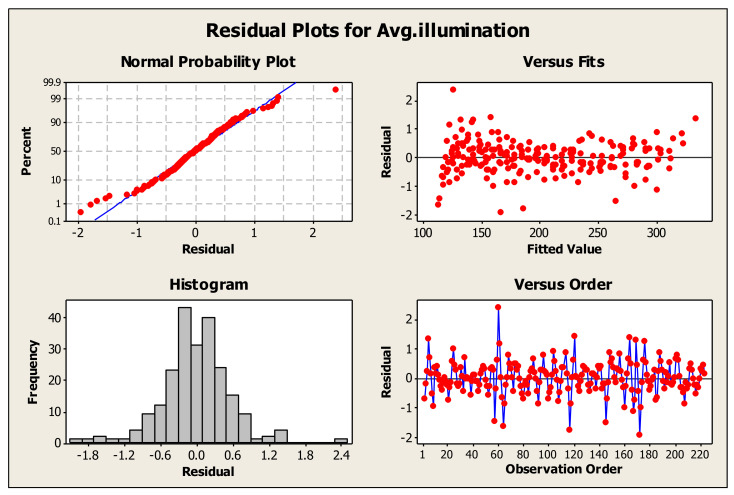
Residual plots for average annual illumination.

Figure 5 represents the residual plots of MANOVA analysis for average annual illuminance as compared to fiber characteristics. The normal probability plot indicates that the experimental values are similar to the statistical expectations, with few outliers. The histogram further characterizes this as the central peak and normal distribution suggest that the data has no skewness or outliers and behave as expected in a normal distribution. The versus fits also demonstrates the suitability of the analysis model used in the study and how it sufficiently represents the obtained data. The observational order indicates the independence of the collected values from one another, eliminating the impact of one value on the next. These residual plots show the suitability of the statistical model and validate the results obtained with MANOVA.

### 3.2. Monthly Variation

The weather conditions, cloud coverage and sun inclination affected the performance and the quantum of transmitted sunlight from month to month. The variants with larger optical fiber areas performed better independent of monthly variation, and luminance peaked around 12.30 p.m. as well.

**Figure 6:**
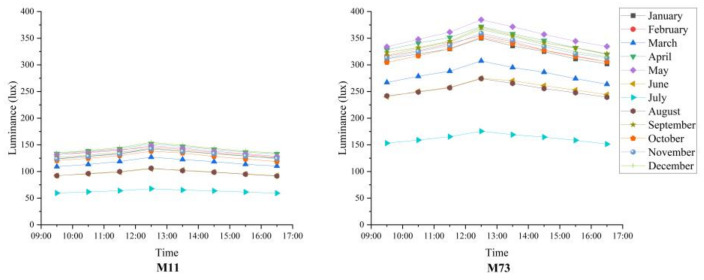
Variation of average monthly luminance vs. time of day for M11 and M73 mixes.

Typical graphs in Figure 6 depict the variation of transmittance based on the month for M11 and M73 mixes and are shown below. M11, having the least fiber volume, exhibits the lowest transmittance, while M73 exhibits the largest transmittance. The best results for all mixes were observed in May, while the worst performance was observed in July.

### 3.3. Seasonal Variation

**Figure 7:**
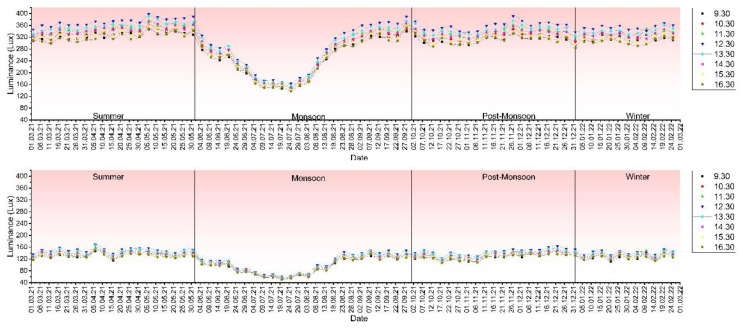
Annual variation of luminance based on the season for M11 and M73 mixes.

When dealing with material dependent on weather, it is crucial to ascertain the behavior relative to the season. The study was focused on the coastal region of Karnataka, India, and the seasonal variation has been considered as per the Indian Metrological Department stipulations. An analysis of the variation of mixes M11 and M73 represents the behavioral trend for all mixes, as these reflect the most significant and smallest transmittance among all variants, as represented in Figure 7. The overcast weather of the monsoon causes a marked drop in the transmitted light, which indicates a higher dependence on artificial luminaries during this period, while the peak transmission occurs in the summer.

Seasonal variation data for various mixes show us that there is a definite visual correlation between the transmittance and percentage fibers as well as the time of the day. The general trend carries through these results as well. Transmittance is reduced in the monsoon and marginally more prominent in the summers.

**Figure 8:**
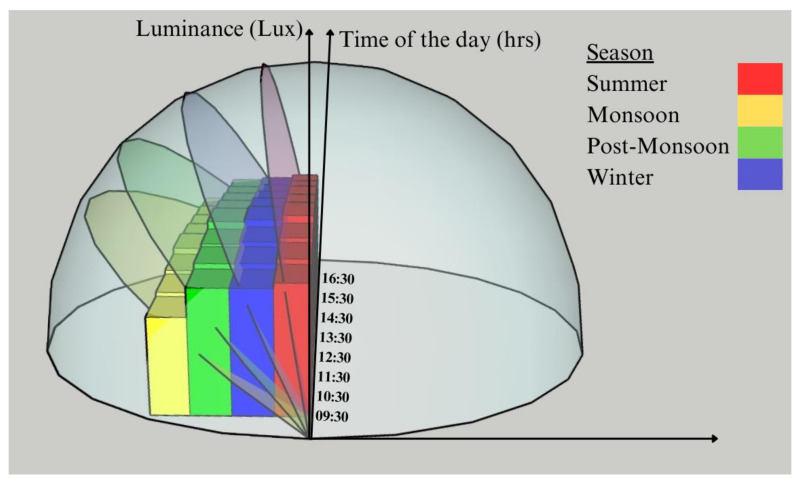
Seasonal illumination concerning the seasonal solar inclination.

Figure 8 illustrates the average seasonal path of the sun throughout the year and the seasonal luminance for mix M73. These angles were obtained from the metrological department of India and then averaged to obtain the seasonal averages. The figure indicates how the sun’s path changes due to seasonal variations and how that significantly affects luminance. This is inherently due to the properties of optical fibers, which have an allowable acceptance cone, and, when the angle of incidence reduces, the photons available for transmittance also reduce.

### 3.4. Applicability of Light-Transmitting Concrete

**Figure 9:**
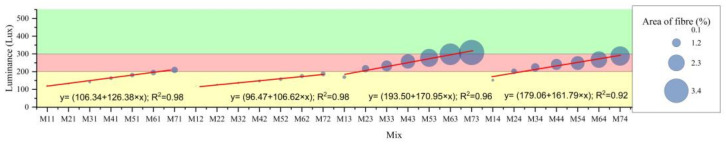
Plot of mix vs. annual average luminance and area of fibers with regression analysis.

Figure 9 represents the average annual illumination of various mixes at peak transmittance, i.e., 12.30 p.m., and an interval classification of these luminances. The use of 0.5 mm fibers, irrespective of the number of fibers or spacing, exhibits transmittance in the 100–200 lux range. This range is suitable for various applications, such as lobbies and common areas, as detailed in Table 4. Most mixes that incorporate 1 mm fibers exhibit transmittance in the 200–300 lux range, which is much more suitable for use in commercial and business spaces where the required light intensity is higher, such as banks, offices and academic establishments. Bundles of seven 1 mm fibers, namely mix M73 and M74, have a luminance of more than 300 lux and can be used in areas where lighting is necessary. These cases are far and few between, such as in deep plan offices or drawing board areas where specialized lighting is essential. While these are possible, having lower fiber ratios for the entire building and enhancing the luminance through artificial luminaries is more practical. Table 4 gives the classification of lighting needs per the Indian standard code for interior illumination [39].

A linear regression model for the relationship between the area of fibers and transmittance is also represented in Figure 9. The model indicates a clear linear relationship between the variables where the area of fibers linearly impacted the transmittance of light, with R-square values ranging between 0.92 and 0.98. This further validates our results, and the hypothesis of a correlation between these two variables is true.

### 3.5. Dependence of Illumination on the Irradiance of Sunlight

**Figure 10:**
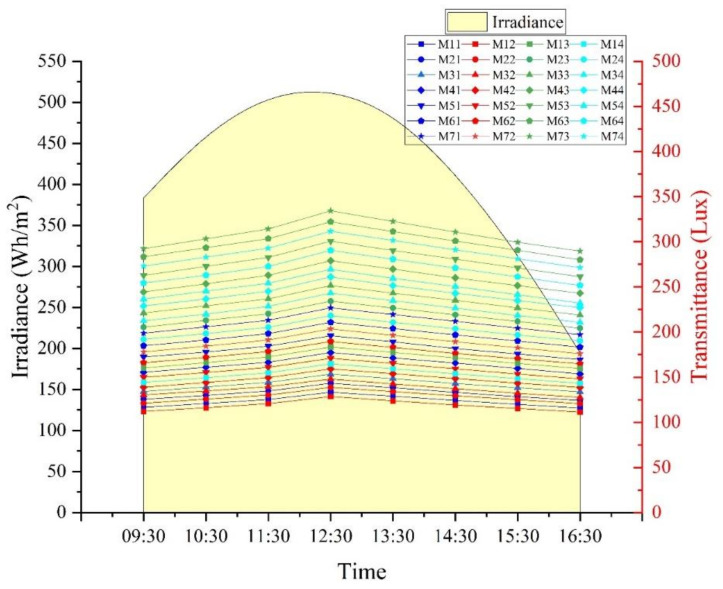
Comparison of the irradiance characteristics and transmittance behavior of mixes.

The irradiance of sunlight is a measure of the light energy of the sun and is an indirect indicator of the intensity of sunlight on the surface of the earth at a specific place. The irradiance data were considered as datum to determine if there was an effect of this on the transmittance of concrete. Irradiance data were obtained for Mangalore International Airport, which is the closest metrological station available to the test location. These details were compared with transmittance, and the results are given in Figure 10.

**Figure 11:**
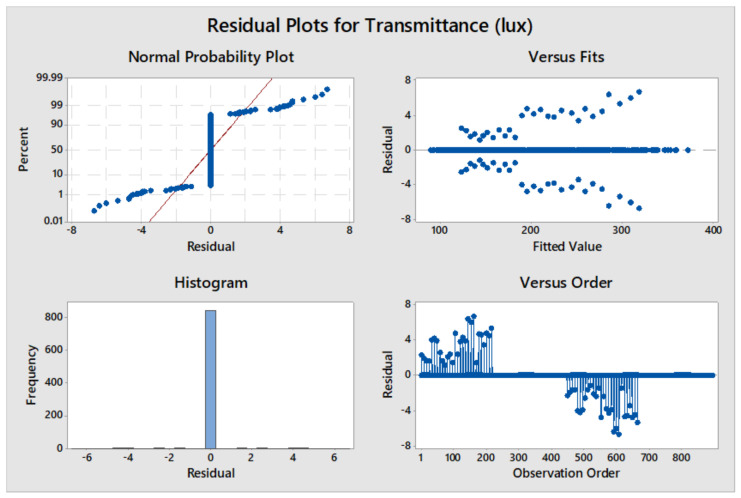
Residual plots of annual illumination compared with irradiance.

There is a strong dependency on the irradiance of the sun in the transmittance of light as their behavior is similar based on the time of the day. This is indicative of an interdependency that was further verified with MANOVA. Table 5 and Figure 11 detail the results of the general linear model and MANOVA analysis. It is clear from the results that there is a definite significance between illumination and irradiance. The same characteristics are seen for interaction effects where the effects analyzed with irradiation have a definite correlation. The spacing of fibers is insignificant for transmitting natural light, and similarly, the interaction between irradiation and spacing is also insignificant. Figure 11 represents the residual plots of MANOVA analysis for average annual illumination as compared to the irradiance characteristics. The plots indicate that the statistical model suitably validates the results obtained with MANOVA.

## 4. Conclusions

This study attempts to better understand the transmittance behavior of light-transmitting concrete with embedded optic fibers in natural sunlight. Based on the experimental and statistical investigation conducted at Manipal Institute of Technology, Manipal, India, these conclusions are derived:

- The correlation between the area of fibers available for transmission and transmittance is evident. As the area of fibers increases, the transmittance also increases. This relationship is independent of the type or nature of the source of illumination. This is further proven when the area and number of fibers are studied, where variants of 1 mm diameter show transmittance up to 85.14% higher than 0.5 mm for the same spacing, just as the variants with a larger number of fibers per bundle exhibited transmittance in the range of 52.33–101.48% for the same spacing.
- The angle of inclination of the sun has an insignificant impact as the scattered light aids in providing enough intensity of light in the directions within the acceptance cone of the fibers, as the acceptance cone of the fibers is as large as 61°.
- Transmittance is highest in the months of April and May, with peak illumination being 360 lux and 372 lux, respectively. The sparse clouds and bright sun cause increased light to be available for transmittance, hence increased transmission occurs.
- The loss of illumination during monsoon is high, and, for most variants, the illuminance is below 300 lux, which indicates the need for artificial illumination during these periods of low light. The transmission ranges for the month of July range between 57 lux and 176 lux, where all the variants have significantly low transmittance in comparison with the other months.
- Most mixes have luminance in the 200–300 lux range, making them suitable for most commercial, public and industrial applications for most months during the year.
- In the present study, considering all the testing parameters and variables, the sample consisting of seven bundled 1 mm fibers with 12 mm spacing is found to have the best performance in light transmission.

A limitation of the study is that the tests use a lux meter; hence, other appropriate testing techniques should be used for larger panels. The study shows a promising prospects of a material that can be used for light transmittance. The bundled fibers have been proven to be relatively more manageable and effective in producing structural light-transmitting concrete. They can provide adequate fiber spacing for aggregates with a higher fiber area for transmittance. Further investigation into the durability and placement techniques is necessary before the material is suitable for integration into the present industrial scenario.

## 5. Patent

Section 2.5 and Figure 3 implement technology filed for a patent as detailed in “Measuring Optical Transmittance of Materials” by Manipal Academy of Higher Education with inventors being Shenoy, A., Nayak, G., Tantri, A. with application number 202341067180, reference number TEMP/E-1/80087/2023-CHE, filed on 6 October 2023 in India.

## Figures and Tables

**Table 1:** Sample designation.

Fiber Diameter		0.5 mm				1 mm			
Fiber Spacing		12 mm		15 mm		12 mm		15 mm	
**Number of fibers**	**1**	M11	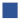	M12	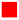	M13	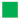	M14	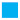
	**2**	M21	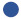	M22	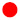	M23	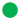	M24	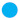
	**3**	M31	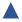	M32	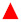	M33	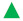	M34	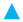
	**4**	M41	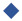	M42	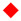	M43	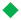	M44	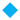
	**5**	M51	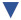	M52	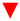	M53	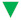	M54	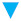
	**6**	M61	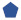	M62	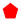	M63	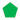	M64	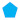
	**7**	M71	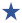	M72	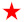	M73	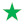	M74	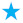

Note: Symbols indicate the symbols used in representations in future graphs.

**Table 2:** Mix proportions in kg/m^3^.

Ordinary Portland Cement	Fly Ash	Coarse Aggregates	Fine Aggregates	Superplasticizer	Water
420	180	780	700	2.514	180

**Table 3:** Results of MANOVA for Average Annual Illumination.

Reference Factor	Average Annual Illumination					
Source	Degree of Freedom	Sequnetial Sums of Squares	Adjusted Sums of Squares	Adjusted Mean Square	F-Statistic Adj. MS/P	Probability
Time	7	18,378.6	18,378.6	2625.5	5059.66	0.000
Diameter	1	403,755.2	403,755.2	403,755.2	778,083.36	0.001
Spacing	1	15,449.2	15,449.2	15,449.2	29,772.47	0.000
No. of fibers	6	270,594.6	270,594.6	45,099.1	86,911.22	0.002
Time × Diameter	7	808.3	808.3	115.5	222.53	0.000
Time × Spacing	7	27.2	27.2	3.9	7.5	0.000
Time × No. of fibers	42	573.9	573.9	13.7	26.33	0.000
Diameter × Spacing	1	485.7	485.7	485.7	935.98	0.003
Diameter × No. of fibers	6	20,932.8	20,932.8	3488.8	6723.34	0.000
Spacing × No. of fibers	6	2847.8	2847.84	74.68	914.68	0.001
Diameter × Spacing × No. of fibers	6	375.9	375.9	62.6	120.73	0.000
Error	133	69.0	69.0	0.5		
Total	233	734,298.3				

S—0.720354, R^2^—99.99%, R^2^ (adj)—99.98%.

**Table 4:** Classification of various light applications based on the range of illumination required [39].

Application	Illumination (lux)	Application	Illumination (lux)	Application	Illumination (lux)
Commerce					
Offices	300	Filing rooms	200	Bank public areas	200
Deep plan general offices	500	Drawing boards	500	Computer and data preparation	300
Computer workstations	300	Print rooms	200	Bank public areas	200
Conference rooms	300	Bank counters	300		
**Supermarkets, hypermarkets**					
General, checkout, showrooms for large objects	300	Shopping precincts, arcades	100		
**Public assembly structures**					
Village halls, worship halls	200	Projection rooms	100	Dressing rooms	200
Cinema hall lobbies	150	Church bodies	100	Vestries	100
Ticket counters	200	Altar, communion table, chancel	100	Organ room	200
Auditorium	50				
**Hotels**					
Entrance halls	50	Cloak rooms	50	Bedrooms	30
Reception, cashier	200	Dining rooms, restaurnats, bars etc	50	Bathrooms	50
**Education**					
General	200	Laboratories	300	Needlework Rooms	300
Lecture and teaching spaces	200	Libraries	200	Sports Halls	200
Seminar Rooms	300	Music Rooms	200	Workshops	200
Art Rooms	300				
**Transport facilities**					
Lounges and waiting areas	150	Timetable	150	Concourse	150
Baggage counter	150	Covered platforms	30	Loading areas	100
Baggage handling	50	Open platforms	20	Customs, immigration	300
**General building areas**					
Entrance halls, lobbies, waiting areas, gatehouses	150	Staff changing, locker and cleaners rooms, cloakrooms, lavatories	50	Resting/waiting areas	100
Lifts, corridors, passageways, stairs	50	Staff restrooms	100	Stores	100
Escalators, travellators	100	Canteens, cafeteria, dining and mess halls	150	Cooking	300
Medical aid treatment rooms	300	Servery and prep	200	Stores and cellars	150
**Car parks-covered**					
Floors	50	Control booth	150	Entrance and exit	50
Ramps and corners	30	Outdoor parks	50		

Note: Colors represent transmittance ranges and suitable mixes as in Figure 9.

**Table 5:** MANOVA results of Average Annual Illumination as compared to irradiance.

RF	Average Annual Illumination				
Source	Degree of Freedom	Adjusted Sums of Squares	Adjusted Mean Square	F-Statistic Adj. MS/P	Probability
Irradiance (Wh/m^2^)	30	559,702	18,657	636.75	0.000
No. of Fibers	6	1,094,366	182,394	6225.09	0.000
Spacing	1	62,684	62,684	2139.40	0.000
Diameter	1	1,633,537	1,633,537	55,752.42	0.000
Irradiance × No. of fibers	180	13,296	74	2.52	0.003
Irradiance × Spacing	30	860	29	0.98	0.525 *
Irradiance × Diameter	30	19,983	666	22.73	0.000
No. of fibers × Spacing	6	11,431	1905	65.03	0.000
No. of fibers × Diameter	6	84,364	14,061	479.89	0.000
Spacing of fibers × Diameter	1	2011	2011	68.63	0.000
Irradiance × No. of fibers × Spacing of fibers	180	215	1	0.04	1.000 *
Irradiance × No. of fibers × Diameter	180	1336	7	0.25	1.000 *
Irradiance × Spacing × Diameter	30	36	1	8.29	1.000 *
No. of fibers × Spacing × Diameter	6	1458	243	8.29	0.000
Irradiance × No. of fibers × Spacing × Diameter	180	317	2	0.06	1.000 *
Error	28	820	29		
Total	895	3,534,472			

S—5.41293, R^2^—99.98%, R^2^ (adj)—99.26%, *—Insignificant.

## Data Availability

Data sharing not applicable.
